# Relationship of Iron Deficiency and Serum Ferritin Levels with Pulmonary Hypertension: The Jackson Heart Study

**DOI:** 10.1371/journal.pone.0167987

**Published:** 2016-12-14

**Authors:** Matthew Jankowich, Beth Elston, Samuel K. Evans, Wen-Chih Wu, Gaurav Choudhary

**Affiliations:** 1 Vascular Research Laboratory, Providence VA Medical Center, Providence, RI, United States of America; 2 Department of Medicine, Alpert Medical School of Brown University, Providence, RI, United States of America; 3 Center for Public Health and Clinical Epidemiology, Brown University, Providence, RI, United States of America; Pennsylvania State University College of Medicine, UNITED STATES

## Abstract

**Purpose:**

Iron deficiency is prevalent in idiopathic pulmonary arterial hypertension (IPAH), but whether iron deficiency or ferritin levels are associated with pulmonary hypertension (PH) in the general population is unknown.

**Methods:**

We performed a cross-sectional analysis of data on iron deficiency (exposure), and PH (pulmonary artery systolic pressure>40mmHg on echocardiogram) (outcome) on subjects with complete data on exposures and outcomes as well as covariates (n = 2,800) enrolled in the Jackson Heart Study, a longitudinal prospective observational cohort study of heart disease in African-Americans from Jackson, Mississippi. Iron deficiency was defined as a serum ferritin level < 15ng/mL (females); < 30ng/mL (males). We determined crude prevalence ratios (PRs) for PH in iron deficient versus non-iron deficient groups using modified Poisson regression modeling. We also analyzed the prevalence of PH by sex-specific quartiles of ferritin (Females ≤ 47ng/mL; > 47ng/mL– 95ng/mL; > 95ng/mL– 171ng/mL; > 171ng/mL; Males ≤ 110ng/mL; > 110ng/mL– 182ng/mL; > 182ng/mL– 294ng/mL; > 294ng/mL), using the same modeling technique with the lowest quartile as the referent.

**Results:**

Median pulmonary artery systolic pressure was 27mmHg (interquartile range 23-31mmHg) in the study cohort. 147 subjects (5.2%) had PH and 140 (5.0%) had iron deficiency. However, of the 147 subjects with PH, only 4 were also iron deficient. The crude PH PR was 0.5 (95% CI 0.2–1.4) in iron-deficiency compared to non-deficient. In analysis by quartiles of ferritin, adjusting for age and sex, there was no evidence of association with PH in quartiles 2 (PR 1.1, 95% CI 0.7–1.6), 3 (PR 0.8, 95% CI 0.5–1.3), or 4 (PR 0.8, 95% CI 0.5–1.2) compared with quartile 1 (referent group, PR 1). Further analyses of the relationship between PH and ferritin as a log-transformed continuous variable or by quartiles of serum iron showed similar results.

**Conclusions:**

In the Jackson Heart Study, the prevalence of PH was similar in iron-deficient and non-iron deficient subjects. There was no evidence of association between ferritin (or serum iron) levels and PH.

**Clinical Implications:**

Iron deficiency has been associated with IPAH, a rare disorder. However, in a large community-based sample of African-Americans, there was no evidence that iron deficiency or low iron levels were associated with PH.

## Introduction

Iron deficiency is the most common nutritional deficiency[1] and the most common cause of anemia worldwide[2]. Iron plays a critical role in oxygen transport and iron depletion affects regulation of hypoxia-inducible factors[3]. In a preclinical study, a reduction in iron and ferritin levels resulted in marked pulmonary vascular remodeling that was reversed by iron supplementation[4]. Similarly, studies in humans have noted that iron augmentation or depletion differentially affect pulmonary vascular responses to hypoxia[5, 6]. Iron deficiency is highly prevalent in cohorts with idiopathic pulmonary arterial hypertension (PAH)[7–10]. Moreover, in patients with PAH and iron deficiency, iron augmentation increased exercise endurance time and aerobic capacity[11]. These preclinical and epidemiological studies suggest that iron may be an important factor in the regulation of pulmonary hemodynamics and that iron deficiency may contribute to the pathogenesis of pulmonary hypertension.

In the U.S., iron deficiency is most common in young females and is more prevalent in African-American women than in whites[1]. Pulmonary hypertension is also prevalent in African-Americans and is more prevalent in African-American females than males[12]. Given that African-Americans are at risk for both iron deficiency and pulmonary hypertension, and that these two conditions may be linked pathogenetically, we therefore set out to determine if iron deficiency was associated with pulmonary hypertension. We examined the association between iron deficiency and pulmonary hypertension, as determined by echocardiographic pulmonary artery systolic pressure measurement, in the largest prospective cohort study of cardiovascular disease in African-Americans, the Jackson Heart Study (JHS).

## Methods

We conducted a cross-sectional analysis of data from the JHS. The conduct of the JHS was approved by the University of Mississippi Medical Center Institutional Review Board. The participants gave written informed consent to participate in the research study. Our analysis of JHS data was reviewed by the Providence VA Medical Center Institutional Review Board and deemed exempt from ongoing review as it involved the study of existing data.

### Population

The JHS is a longitudinal population-based cohort study of cardiovascular disease which recruited noninstitutionalized adult participants residing in Jackson, MS who self-identified as African-Americans[13]. Subjects answered predefined questionnaires and underwent venipuncture, including measurements of iron indices, and echocardiography at the time of first/baseline exam in 2000–2004. The cohort used for the current study included participants that had available ferritin and iron levels as well as measureable tricuspid regurgitant (TR) jet velocity on echocardiography (allowing for estimation of the pulmonary artery systolic pressure), and covariate measurements (detailed below) at the time of the first study visit. Participants were excluded if they lacked an iron or ferritin level, lacked a measureable TR jet, or lacked covariate measurements. Participants with a c-reactive protein level greater than 10mg/dl suggestive of an acute inflammatory process were also excluded.

### Exposure

The main exposure was iron deficiency. We examined iron deficiency using both serum ferritin and iron levels as markers. A complete description of the specimen collection procedures and quality control measures for the Jackson Heart Study is available online.[14] Blood for serum iron and ferritin levels was drawn at the baseline study visit from the antecubital fossa of supine subjects who had been fasting by trained technicians. Serum ferritin was measured in nanograms/milliliter (ng/ml) using Roche immunoturbimetric assay[15]. The assay’s accuracy based on recovery criteria has been reported to be within ± 4μg/L (≤ 8.99 pmol/L, ≤ 4ng/mL) of the initial value for samples with a ferritin value ≤ 40μg/L (≤ 89.9 pmol/L, ≤ 40 ng/mL) and within ± 10% for samples with a ferritin value> 40μg/L[16]. Serum iron was measured in micrograms/deciliter (ug/dL) using Roche FerroZine colorimetric assay[15]. The assay was standardized to NIST traceable iron standards and calibrated against control sera from the manufacturer[15].

Iron deficiency was defined based on sex-specific cut-offs with deficiency defined using serum ferritin values as follows: female <15ng/ml; males <30ng/ml, based on Jackson Heart Study reference range. Participants were also divided into sample, sex specific quartiles of ferritin with the following cutoffs: Females ≤ 47ng/mL; > 47ng/mL– 95ng/mL; > 95ng/mL– 171ng/mL; > 171ng/mL; Males ≤ 110ng/mL; > 110ng/mL– 182ng/mL; > 182ng/mL– 294ng/mL; > 294ng/mL.

In separate analyses, iron deficiency was alternatively defined using serum iron levels based on sex-specific cut-offs with deficiency defined as follows: females <30ug/dl; males < 45ug/dl, based on Jackson Heart Study reference range. Additional analyses were conducted with the participants divided into sample, sex specific quartiles by iron level with the following cutoffs: ≤ 57μg/dL; > 57μg/dL– 73μg/dL; > 73μg/dL– 90μg/dL; > 90μg/dL; Males: ≤ 68μg/dL; > 68μg/dL– 84μg/dL; > 84μg/dL– 103μg/dL; > 103μg/dL.

Additionally, we also conducted sensitivity analyses using continuous measures of ferritin and iron levels. Ferritin was naturally log-transformed for the continuous analysis to approximate normality.

### Outcome

The main outcome was presence of pulmonary hypertension, defined as a pulmonary artery systolic pressure>40mmHg on baseline echocardiography.

Detailed echocardiography procedures are available online[17]. Briefly, echocardiograms were recorded by trained sonographers and interpreted by experienced cardiologists in a standardized manner at the University of Mississippi Medical Center.[17] Standard echocardiographic views were obtained and measurements performed by the interpreting physician who was blinded to the participants’ clinical data. The pulmonary artery systolic pressure was calculated by addition of 5mmHg right atrial pressure to the transtricuspid gradient[18].

### Clinical covariates

In addition to age (<55, 55-<65, ≥65 years) and sex (male, female), the following covariates were utilized in the statistical models detailed below: body mass index was categorized by American Heart Association ideal cardiovascular health categorization (poor health: BMI≥30kg/m^2^; intermediate health: BMI≥25, but <30kg/m^2^; ideal health: BMI<25kg/m^2^)[19]. Pulse pressure (in mmHg) was defined as the difference between systolic and diastolic blood pressures. Systemic hypertension was defined as systolic blood pressure ≥140mmHg or diastolic blood pressure≥90mmHg or if the subject was using blood pressure lowering medications[20]. Diabetes was considered present if hemoglobin A1C was ≥6.5%, if a fasting plasma glucose was ≥126mg/dl, or if use of diabetes medications was reported[21]. Coronary heart disease was considered present if the subject reported a history of coronary heart disease, a prior abnormal stress test, prior coronary bypass graft, or prior coronary angioplasty, or if there was EKG evidence of a prior myocardial infarction (per Minnesota code). History of chronic lung disease was considered present if the subjects responded to the question “Has your doctor or health professional ever said you have chronic lung disease, such as bronchitis or emphysema?” in the affirmative at baseline study visit. Spirometry profiles were categorized into normal, obstructive and restrictive. A normal spirometry profile was defined as an FEV1/FVC ratio≥0.70 and an FVC≥80% predicted. An obstructive spirometry profile was defined as an FEV1/FVC ratio<0.70. A restrictive spirometry profile was defined as an FEV1/FVC ratio≥0.70 with an FVC<80% predicted. Percent predicted values for FVC were derived from NHANES III data[22]. Ejection fraction was dichotomized into <50% and ≥ 50%. Hemoglobin was measured in grams/deciliter and the level of high sensitive C-reactive protein level was measured in milligrams per deciliter.

### Statistical analysis

We examined the association of iron deficiency with pulmonary hypertension using modified Poisson regression modeling with a log-link function and robust variance estimator to obtain prevalence ratios (PR). To analyze the relationship between iron deficiency and pulmonary hypertension, we performed analyses for binary, quartile and continuous versions of the exposure measurements (ferritin and iron levels).

In the binary analysis, the presence of pulmonary hypertension in iron deficiency (based on low ferritin or low iron levels) was contrasted with pulmonary hypertension in participants with non-deficient levels of ferritin or iron. We further examined prevalence ratios for pulmonary hypertension by quartiles of ferritin or iron levels, contrasting exposure quartiles 2, 3, and 4 with quartile 1, the referent group. We also analyzed continuous measurements of log-ferritin or iron with results expressed as prevalence ratios for pulmonary hypertension per 10% change in ferritin and 10ug/dl change in iron, respectively. For the quartile and continuous outcome analyses, in addition to crude analyses, we also obtained adjusted prevalence ratios for three separate models. The first model adjusted for age and sex. The second model included all covariates listed in the above section: age, sex, body mass index category, pulse pressure, hypertension, diabetes, coronary heart disease, history of chronic lung disease, spirometry profile and ejection fraction. The second model was adapted from a model of pulmonary hypertension adapted from Choudhary et al[12]. The adaptation in this model from the originally published model was the inclusion of an ejection fraction less than 50%. A third exploratory analysis was performed further adjusting the second model for hemoglobin and for C-reactive protein.

Analyses were performed using SAS 9.4 (Cary, NC). A two-sided p value less than 0.05 was considered significant.

## Results

**Fig 1:**
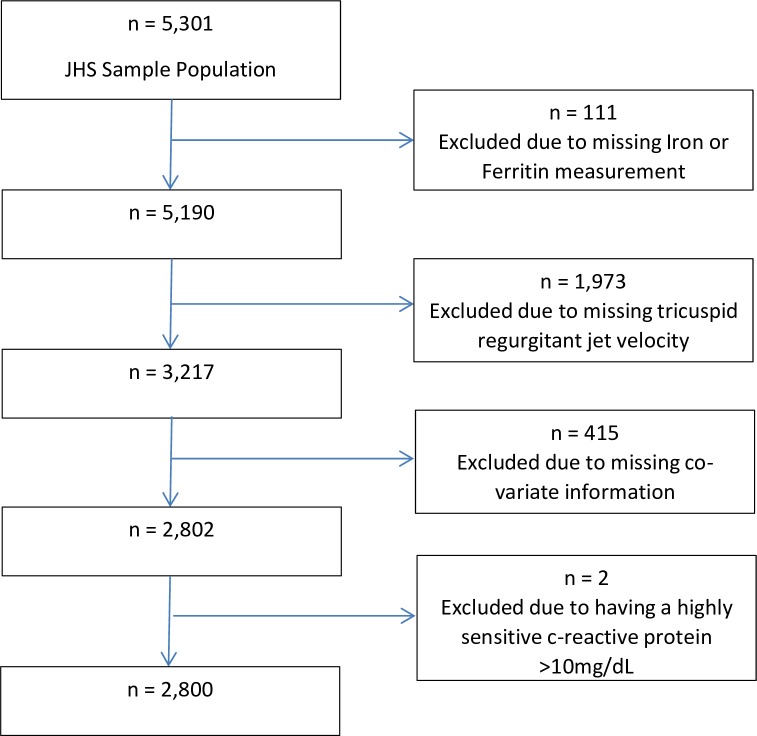
Details of analysis sample derivation.

From the JHS cohort (n = 5,301), one hundred and eleven participants were excluded due to missing iron or ferritin measurement, 1,973 were excluded due to missing TR jet, 415 were excluded due to missing covariate data, and an additional two were excluded due to having a highly sensitive c-reactive protein >10 mg/dL (Fig 1). The details of those with complete information (n = 2,800) versus those excluded due to missing outcome (pulmonary artery systolic pressure) or exposure (ferritin or iron level) and those excluded due to missing co-variates or c-reactive protein >10mg/dL are detailed in S1 Table.

**Fig 2:**
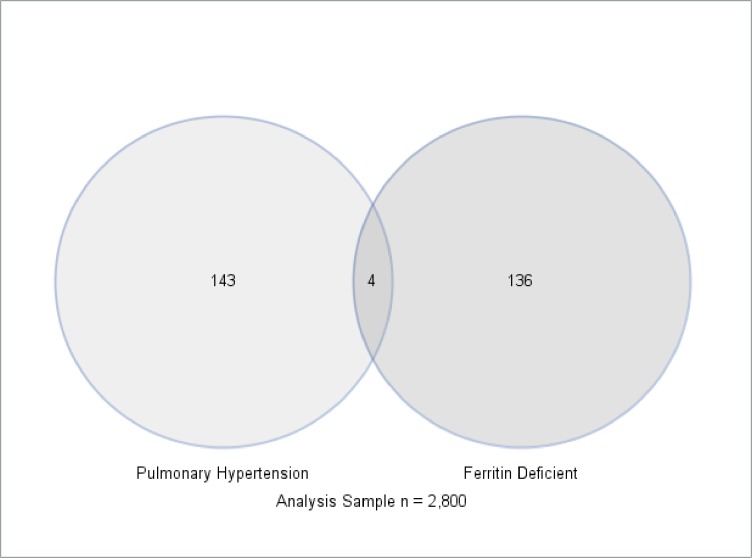
Venn diagram of pulmonary hypertension (n = 147) and iron deficiency based on low ferritin levels (n = 140) in the Jackson Heart Study cohort. Only 4 participants had both iron deficiency and pulmonary hypertension.

S1 Table shows the baseline characteristics of the cohort used for the present analysis. Of 2,800 with complete information, 140 (5.0%) had iron deficiency based on ferritin levels, while 92 (3.3%) had iron deficiency based on iron levels. The median (interquartile range) pulmonary artery systolic pressure in the study cohort was 27mmHg (23-31mmHg). One hundred and forty seven participants (5.2%) had pulmonary hypertension. However, only 4 had both iron deficiency, based on serum ferritin level, and pulmonary hypertension (2.7% of those with pulmonary hypertension), while only 7 had iron deficiency, based on serum iron level, and pulmonary hypertension (4.8% of those with pulmonary hypertension) (Fig 2, S2 Table). The crude prevalence ratio for PH for those with iron deficiency based on low ferritin level was 0.5 (95% confidence interval 0.2–1.4) and based on low iron level was 1.5 (95% confidence interval 0.7–3.1) (S2 Table).

The baseline characteristics of the study cohort, stratified by quartiles of ferritin, are shown in Table 1. Participants in the highest quartile of ferritin were more likely to be in a middle-aged or older age group, to have a BMI≥30kg/m^2^, and to have hypertension, diabetes, and a higher pulse pressure and higher c-reactive protein levels compared to the lowest quartile. However, spirometry profiles and proportions of participants with a decreased left ventricular ejection fraction were similar across the quartiles. Of note, hemoglobin was lowest in the lowest quartile of ferritin, while C-reactive protein was highest in the highest quartile.

**Table 1:** Baseline demographics of the analysis sample and stratified by quartiles of ferritin.

Characteristic	Analysis Samplen (%)	Quartile 1 n = 728 (Ferritin ≤ 47ng/mL females, ≤ 110ng/mL males)n (%)	Quartile 2 n = 728 (Ferritin > 47ng/mL– 95ng/mL females, > 110ng/mL– 182ng/mL males)n (%)	Quartile 3 n = 686 (Ferritin > 95ng/mL– 171ng/mL females, > 182ng/mL– 294ng/mL males)(n%)	Quartile 4 n = 734 (Ferritin >171ng/mL females, >294ng/mL males)n (%)	P values for comparison across quartiles
**Total**	2,800	712	714	665	709	
**Iron Quartile**^**a**^						<0.001
**Q1**	675 (24.1)	290 (40.7)	139 (19.5)	135 (20.3)	111 (15.7)	
**Q2**	695 (24.8)	140 (19.7)	197 (27.6)	179 (26.9)	179 (25.2)	
**Q3**	705 (25.2)	121 (17.0)	182 (25.5)	174 (26.2)	228 (32.2)	
**Q4**	725 (25.9)	161 (22.6)	196 (27.5)	177 (26.6)	191 (26.9)	
**Iron (μg/dL)**^**b**^	77 (61, 95)	68.0 (50.0, 92.0)	79.0 (64.0, 96.0)	79.0 (63.0, 97.0)	81.0 (67.0, 96.0)	<0.001
**Male**						0.66
**No**	1,882 (67.2)	468 (65.7)	490 (68.6)	443 (66.6)	481 (67.8)	
**Yes**	918 (32.8)	244 (34.3)	224 (31.4)	222 (33.4)	228 (32.2)	
**Age (years)**						<0.001
**< 55**	1,282 (45.8)	451 (63.3)	333 (46.6)	268 (40.3)	230 (32.4)	
**55 - < 65**	811 (29.0)	119 (16.7)	196 (27.5)	222 (33.4)	274 (38.6)	
**≥ 65**	707 (25.3)	142 (19.9)	185 (25.9)	175 (26.3)	205 (28.9)	
**BMI Health**						<0.001
**Poor**	1,425 (50.9)	322 (45.2)	346 (48.5)	360 (54.1)	397 (56.0)	
**Intermediate**	958 (34.2)	251 (35.3)	264 (37.0)	208 (31.3)	235 (33.1)	
**Ideal**	417 (14.9)	139 (19.5)	104 (14.6)	97 (14.6)	77 (10.9)	
**Pulse Pressure (mm Hg)**^**b**^	45 (37, 56)	43.0 (36.0, 54.0)	44.0 (37.0, 54.0)	46.0 (37.0, 58.0)	47.0 (39.0, 57.0)	<0.001
**Hypertension**						<0.001
**No**	1,171 (41.8)	358 (50.3)	317 (44.4)	265 (39.8)	231 (32.6)	
**Yes**	1,629 (58.2)	354 (49.7)	397 (55.6)	400 (60.2)	478 (67.4)	
**Diabetes**						<0.001
**No**	2,272 (81.1)	636 (89.3)	583 (81.7)	539 (81.1)	514 (72.5)	
**Yes**	528 (18.9)	76 (10.7)	131 (18.3)	126 (18.9)	195 (27.5)	
**Coronary Heart Disease**						0.67
**No**	2,514 (89.8)	632 (88.8)	648 (90.8)	597 (89.8)	637 (89.8)	
**Yes**	286 (10.2)	80 (11.2)	66 (9.2)	68 (10.2)	72 (10.2)	
**History of Chronic Lung Disease**						0.74
**No**	2,612 (93.3)	663 (93.1)	672 (94.1)	620 (93.2)	657 (92.7)	
**Yes**	188 (6.7)	49 (6.9)	42 (5.9)	45 (6.8)	52 (7.3)	
**Spirometry Profile**						0.79
**Normal**	2,004 (71.6)	521 (73.2)	517 (72.4)	472 (71.0)	494 (69.7)	
**Obstructive**	244 (8.7)	63 (8.8)	60 (8.4)	57 (8.6)	64 (9.0)	
**Restrictive**	552 (19.7)	128 (18.0)	137 (19.2)	136 (20.5)	151 (21.3)	
**Left Ventricle Ejection Fraction (< 50%)**						0.32
**No**	2,727 (97.4)	691 (97.1)	701 (98.2)	643 (96.7)	692 (97.6)	
**Yes**	73 (2.6)	21 (2.9)	13 (1.8)	22 (3.3)	17 (2.4)	
**Hemoglobin (g/dL)**^**b**^	13 (12, 14)	12.7 (11.7, 13.6)	13.0 (12.2, 13.9)	13.0 (12.2, 13.9)	13.1 (12.3, 14.0)	<0.001
**Highly Sensitive C-Reactive Protein (mg/dL)**^**b**^	0.3 (0.1, 0.5)	0.22 (0.09, 0.50)	0.25 (0.09, 0.52)	0.26 (0.11, 0.54)	0.29 (0.13, 0.59)	<0.001

^a^ Females: Quartile 1 ≤ 57μg/dL; Quartile 2 > 57μg/dL– 73μg/dL; Quartile 3 > 73μg/dL– 90μg/dL; Quartile 4 > 90μg/dL

Males: Quartile 1 ≤ 68μg/dL; Quartile 2 > 68μg/dL– 84μg/dL; Quartile 3 > 84μg/dL– 103μg/dL; Quartile 4 > 103μg/dL

^b^ Median (Quartile 1, Quartile 3)

We found no evidence of association in age- and sex-adjusted prevalence ratios for pulmonary hypertension across the quartiles of ferritin (Table 2). For example, the prevalence ratio for PH in the highest quartile of ferritin was 0.8 (95% CI 0.5–1.2), not significantly different than the lowest quartile. Further adjustment for factors in our second and third pulmonary hypertension models did not affect these results. Analyses based on quartiles of iron showed similar results.

**Table 2:** Association of Ferritin and Iron with Pulmonary Hypertension in the JHS analysis sample, n = 2,800.

	Model 1^a^ PR (95% CL)	Model 2^b^ PR (95% CL)	Model 3^c^ PR (95% CL)
	**Ferritin**		
**Continuous**^**d**^	1.0 (1.0, 1.0)	1.0 (1.0, 1.0)	1.0 (1.0, 1.0)
**Quartile**^**e**^			
**1**	1 (ref)	1 (ref)	1 (ref)
**2**	1.1 (0.7, 1.6)	1.1 (0.7, 1.7)	1.1 (0.7, 1.8)
**3**	0.8 (0.5, 1.3)	0.8 (0.5, 1.3)	0.8 (0.5, 1.3)
**4**	0.8 (0.5, 1.2)	0.8 (0.5, 1.3)	0.8 (0.5, 1.4)
	**Iron**		
**Continuous**^**f**^	0.9 (0.9, 1.0)	1.0 (0.9, 1.0)	1.0 (0.9, 1.1)
**Quartile**^**g**^			
**1**	1 (ref)	1 (ref)	1 (ref)
**2**	0.7 (0.5, 1.1)	0.8 (0.5, 1.3)	0.9 (0.6, 1.4)
**3**	0.7 (0.5, 1.1)	0.8 (0.6, 1.3)	0.9 (0.6, 1.5)
**4**	0.7 (0.5, 1.1)	1.0 (0.7, 1.5)	1.1 (0.7, 1.8)

^a^ Adjusted for age and sex

^b ^Adjusted for age, sex, BMI, pulse pressure, hypertension, diabetes, coronary heart disease, history of chronic lung disease, spirometry profile, left ventricle ejection fraction

^c ^Adjusted for age, sex, BMI, pulse pressure, hypertension, diabetes, coronary heart disease, history of chronic lung disease, spirometry profile, left ventricle ejection fraction, hemoglobin, highly sensitive c-reactive protein

^d^Prevalence Ratio expressed per 10% increase ln ferritin

^e^ Ferritin Females: Quartile 1 ≤ 47ng/mL; Quartile 2 > 47ng/mL– 95ng/mL; Quartile 3 > 95ng/mL– 171ng/mL; Quartile 4 > 171ng/mL

Ferritin Males: Quartile 1 ≤ 110ng/mL; Quartile 2 > 110ng/mL– 182ng/mL; Quartile 3 > 182ng/mL– 294ng/mL; Quartile 4 > 294ng/mL

^f ^Prevalence Ratio expressed per 10μg/dL increase in iron

^g ^Iron Females: Quartile 1 ≤ 57μg/dL; Quartile 2 > 57μg/dL– 73μg/dL; Quartile 3 > 73μg/dL– 90μg/dL; Quartile 4 > 90μg/dL

Iron Males: Quartile 1 ≤ 68μg/dL; Quartile 2 > 68μg/dL– 84μg/dL; Quartile 3 > 84μg/dL– 103μg/dL; Quartile 4 > 103μg/dL

Further analysis of the relationship between ferritin or iron levels as continuous variables and pulmonary hypertension showed no evidence of an association between PH and serum ferritin or iron levels. For example, for every 10% increase in ferritin, the age- and sex-adjusted prevalence ratio for PH was 1.0 (95% confidence interval 1.0–1.0) (Table 2).

## Discussion

In the current study, we found that iron deficiency, based on low serum ferritin or iron levels, was present in only a small number of participants in the Jackson Heart Study with pulmonary hypertension by echocardiogram. We found no evidence of association between iron deficiency or low iron/ferritin levels and pulmonary hypertension.

While our study is the first, to our knowledge, to report on the association between iron deficiency and pulmonary hypertension in a cohort from the general population, a smaller prior study by Altintas et al examined the prevalence of pulmonary hypertension in a group of patients with essential thrombocythemia (n = 46) in comparison to a control group with iron deficiency and resultant reactive thrombocytosis (n = 40)[23]. Pulmonary hypertension, defined as an estimated right ventricular systolic pressure>35mmHg in this study, was observed in 22/46 (48%) with essential thrombocythemia, but in 0/40 (0%) with iron deficiency and reactive thrombocytosis[23]. Our results support this study’s finding that there is no evidence that pulmonary hypertension is a characteristic of iron deficiency.

A recently published study by Frise et al showed that while there was no difference in pulmonary artery systolic pressure in 13 subjects with iron deficiency and a group of age- and sex-matched controls without iron deficiency under normoxic conditions, the iron deficient group had a greater rise in PASP following hypoxic exposure as compared to the iron replete group[24]. Furthermore, infusion of intravenous iron attenuated the rise in PASP with hypoxia in both groups, but the magnitude of attenuation was greater in the iron deficient group[24]. This study indicates that iron may play an important role in the hypoxic pulmonary vascular response. The cohort we studied underwent measurement of PASP in Jackson, MS, close to sea level. Therefore, our finding of a lack of evidence of association of iron deficiency with pulmonary hypertension under near sea level conditions is consistent with Frise et al’s findings during normoxia. However, there is a possibility that iron deficiency could be associated with pulmonary hypertension in setting of hypoxia, such as high altitude residents or visitors.

Our results are in contrast to prior human studies showing an elevated prevalence of iron deficiency in patients with pulmonary arterial hypertension, including patients with both idiopathic[7–9] and heritable pulmonary hypertension[9]. Since the JHS population was recruited from the community population and has a high burden of cardiopulmonary comorbidities, the majority of PH observed in this cohort is likely non-PAH PH. Therefore, our data suggests that, in contrast to what has been observed in PAH cohorts, other forms of pulmonary hypertension may not show evidence of association with iron deficiency or iron/ ferritin levels.

This interpretation is supported by results from some other studies as well. In a study by Soon et al, iron deficiency was prevalent in patients with idiopathic pulmonary arterial hypertension (point prevalence of iron deficiency of 30.1%), especially pre-menopausal women (point prevalence of iron deficiency of 50.0%), but the prevalence of iron deficiency in a control group with chronic thromboembolic pulmonary hypertension was only 4.9%[9]. This prevalence estimate is similar to what is described in the general population[1] and was similar to the prevalence of iron deficiency in the JHS cohort at 5.0% (by ferritin level). Similarly, another study in chronic thromboembolic pulmonary hypertension patients found no difference in iron parameters between patients with chronic thromboembolic pulmonary hypertension and controls[25]. In a study examining zinc-protoporphyrin to heme ratio as a marker of iron deficiency or altered iron metabolism, a high zinc-protoporphyrin to heme ratio was noted in idiopathic pulmonary arterial hypertension patients, and this ratio was related to disease severity, but a similarly high ratio was not observed in associated pulmonary arterial hypertension of various causes[26]. These studies suggest that iron deficiency or iron-handling abnormalities are not universal to all forms of pulmonary hypertension, but may be particular to idiopathic or heritable pulmonary arterial hypertension.

Our study does not support a straightforward direct mechanistic association between iron deficiency and pulmonary hypertension in humans as observed in preclinical studies. In a rat model utilizing adult Sprague-Dawley rats, rats exposed to an iron deficient diet developed low serum and tissue iron levels and anemia. In this model, dietary iron deficiency was sufficient to induce elevations in pulmonary arterial pressure and pulmonary vascular resistance by itself, as well as histological evidence of pulmonary vascular remodeling[4]. However, we did not find any association between iron deficiency and low iron and ferritin levels with presence of pulmonary hypertension. This may be related to severity or acuity of iron deficiency induced in the animal models, or species-specific differences in response of the pulmonary circulation to iron deficiency.

Our study has several limitations. Being cross-sectional in nature, we cannot determine the length of time participants had iron deficiency; if there is a relationship between pulmonary hypertension and iron deficiency, sufficient time in an iron deficient state may be needed to allow development of elevated pulmonary artery pressures. In idiopathic pulmonary arterial hypertension patients, iron deficiency may be a marker for other abnormalities, such as impaired regulation of hypoxia-inducible factors or inflammation that contribute to pulmonary hypertension[3]. We could not assay factors such as hypoxia-inducible factor that may be important in the pathogenesis of pulmonary hypertension in iron-dysregulated states. Right heart catheterization was not performed in the study population, and echocardiographic estimates of pulmonary artery systolic pressure may have resulted in categorization of patients as having pulmonary hypertension who actually had normal pulmonary artery pressures, potentially biasing our results. However, we did note an elevated prevalence of pulmonary hypertension in association with risk factors for pulmonary hypertension such as airflow obstruction.

In summary, in a large community-based sample of African-Americans, there was no evidence that iron deficiency or low iron levels were associated with pulmonary hypertension.

## Supporting Information

S1 Table Characteristics of the analysis sample and excluded participants. (DOCX)

S2 Table Demographics of individuals with pulmonary hypertension in the analysis sample. (DOCX)
