# Supplementary material for: Relationship of Iron Deficiency and Serum Ferritin Levels with Pulmonary Hypertension: The Jackson Heart Study
Source: PLoS One. 2016 Dec 14;11(12):e0167987. doi: 10.1371/journal.pone.0167987 (PMC5156429; doi:10.1371/journal.pone.0167987)
Supplement: S1 Table — (DOCX) [file pone.0167987.s001.docx]

**S1 Table. Characteristics of the analysis sample and excluded participants.**

| **Characteristic** | **Analysis Sample n (%)*** | **Excluded due to missing outcome of exposure information n (%)*** | **Excluded due to missing co-variate information or HSCRP > 10mg/dL n (%)*** |
| --- | --- | --- | --- |
| **Total** | 2,800 | 2,084 | 417 |
| **Pulmonary Hypertension** |  |  |  |
| **No** | 2,653 (94.8) | 61 (93.8) | 384 (92.1) |
| **Yes** | 147 (5.3) | 4 (6.2) | 33 (7.9) |
| **Pulmonary Artery systolic pressure (mm Hg)^a^** | 27 (23, 31) | 28 (24, 34) | 28 (24, 34) |
| **Iron Deficient by Ferritin^b^** |  |  |  |
| **No** | 2,660 (95.0) | 1,872 (94.8) | 404 (96.9) |
| **Yes** | 140 (5.0) | 101 (5.1) | 13 (3.1) |
| **Ferritin Quartile^c^** |  |  |  |
| **Q1** | 712 (25.4) | 508 (25.7) | 90 (21.6) |
| **Q2** | 714 (25.5) | 490 (24.8) | 103 (24.7) |
| **Q3** | 665 (23.8) | 509 (25.8) | 107 (25.7) |
| **Q4** | 709 (25.3) | 466 (23.6) | 117 (28.1) |
| **Ferritin (ηg/mL)^a^** | 115 (61, 215) | 130 (64, 231) | 122 (76, 214) |
| **Iron Deficient by Iron^d^** |  |  |  |
| **No** | 2,708 (96.7) | 1,896 (96.1) | 407 (97.6) |
| **Yes** | 92 (3.3) | 77 (3.9) | 10 (2.4) |
| **Iron Quartile^e^** |  |  |  |
| **Q1** | 675 (24.1) | 540 (27.4) | 113 (27.1) |
| **Q2** | 695 (24.8) | 492 (24.9) | 118 (28.3) |
| **Q3** | 705 (25.2) | 454 (23.0) | 111 (26.6) |
| **Q4** | 725 (25.9) | 487 (24.7) | 75 (18.0) |
| **Iron (µg/dL)^a^** | 77 (61, 95) | 77 (60, 97) | 74 (60, 88) |
| **Male** |  |  |  |
| **No** | 1,882 (67.2) | 1,200 (57.6) | 285 (68.3) |
| **Yes** | 918 (32.8) | 884 (42.4) | 132 (31.7) |
| **Age (years)** |  |  |  |
| **< 55** | 1,282 (45.8) | 1,153 (55.3) | 127 (30.5) |
| **55 - < 65** | 811 (29.0) | 494 (23.7) | 127 (30.5) |
| **≥ 65** | 707 (25.3) | 437 (21.0) | 163 (39.1) |
| **BMI Health** |  |  |  |
| **Poor** | 1,425 (50.9) | 1,177 (56.6) | 220 (53.1) |
| **Intermediate** | 958 (34.2) | 618 (29.7) | 125 (30.2) |
| **Ideal** | 417 (14.9) | 283 (13.6) | 69 (16.7) |
| **Pulse Pressure (mm Hg)^a^** | 45 (37, 56) | 45 (36, 56) | 49 (40, 60) |
| **Hypertension** |  |  |  |
| **No** | 1,171 (41.8) | 816 (39.2) | 122 (29.3) |
| **Yes** | 1,629 (58.2) | 1,265 (60.8) | 294 (70.7) |
| **Diabetes** |  |  |  |
| **No** | 2,272 (81.1) | 1,497 (73.9) | 319 (76.9) |
| **Yes** | 528 (18.9) | 528 (26.1) | 96 (23.1) |
| **Coronary Heart Disease** |  |  |  |
| **No** | 2,514 (89.8) | 1,770 (89.2) | 218 (84.5) |
| **Yes** | 286 (10.2) | 214 (10.8) | 40 (15.5) |
| **History of Chronic Lung Disease** |  |  |  |
| **No** | 2,612 (93.3) | 1,920 (92.5) | 373 (91.0) |
| **Yes** | 188 (6.7) | 156 (7.5) | 37 (9.0) |
| **Spirometry Profile** |  |  |  |
| **Normal** | 2,004 (71.6) | 1,317 (68.7) | 167 (65.7) |
| **Obstructive** | 244 (8.7) | 164 (8.6) | 29 (11.4) |
| **Restrictive** | 552 (19.7) | 437 (22.8) | 58 (22.8) |
| **Left Ventricle Ejection Fraction (< 50%)** |  |  |  |
| **No** | 2,727 (97.4) | 1,775 (95.9) | 385 (96.7) |
| **Yes** | 73 (2.6) | 75 (4.1) | 13 (3.3) |
| **Hemoglobin (g/dL)^a^** | 13 (12, 14) | 13 (12, 14) | 13 (12, 14) |
| **Highly Sensitive C-Reactive Protein (mg/dL)^a^** | 0.3 (0.1, 0.5) | 0.3 (0.1, 0.6) | 0.3 (0.1, 0.6) |

* Except where noted.

^a^ Median (Quartile 1, Quartile 3)

^b^ Iron deficient by ferritin <15ng/ml (females); <30ng/ml (males)

^c^ Females: Quartile 1 ≤ 47ng/mL; Quartile 2 > 47ng/mL – 95ng/mL; Quartile 3 > 95ng/mL – 171ng/mL; Quartile 4 > 171ng/mL

Males: Quartile 1 ≤ 110ng/mL; Quartile 2 > 110ng/mL – 182ng/mL; Quartile 3 > 182ng/mL – 294ng/mL; Quartile 4 > 294ng/mL

^d^ Iron deficient by iron <30ug/dl (female); < 45ug/dl (males)

^e^ Females: Quartile 1 ≤ 57µg/dL; Quartile 2 > 57µg/dL – 73µg/dL; Quartile 3 > 73µg/dL – 90µg/dL; Quartile 4 > 90µg/dL

Males: Quartile 1 ≤ 68µg/dL; Quartile 2 > 68µg/dL – 84µg/dL; Quartile 3 > 84µg/dL – 103µg/dL; Quartile 4 > 103µg/dL
