# Supplementary material for: Relationship of Iron Deficiency and Serum Ferritin Levels with Pulmonary Hypertension: The Jackson Heart Study
Source: PLoS One. 2016 Dec 14;11(12):e0167987. doi: 10.1371/journal.pone.0167987 (PMC5156429; doi:10.1371/journal.pone.0167987)
Supplement: S2 Table — (DOCX) [file pone.0167987.s002.docx]

**S2 Table. Demographics of individuals with pulmonary hypertension in the analysis sample.**

| **Characteristic** | **n (%)** | **Crude Prevalence Ratio (95 % Confidence Limits)** |
| --- | --- | --- |
| **Total** | 147 |  |
|  |  |  |
| **Pulmonary Artery Systolic Pressure (mm Hg) ^a^** | 44 (42, 49) |  |
| **Ferritin Deficiency^b^** |  |  |
| **No** | 143 (97.3) | 1 (ref) |
| **Yes** | 4 (2.7) | 0.5 (0.2, 1.4) |
| **Ferritin Quartiles^c^** |  |  |
| **Q1** | 31 (21.1) | 1 (ref) |
| **Q2** | 44 (29.9) | 1.4 (0.9, 2.2) |
| **Q3** | 34 (23.1) | 1.2 (0.7, 1.9) |
| **Q4** | 38 (25.9) | 1.2 (0.8, 2.0) |
| **Ferritin (ng/mL)^a,d^** | 103 (69, 199) | 1.0 (1.0, 1.0) |
| **Iron Deficiency^e^** |  |  |
| **No** | 140 (95.2) | 1 (ref) |
| **Yes** | 7 (4.8) | 1.5 (0.7, 3.1) |
| **Iron Quartiles^f^** |  |  |
| **Q1** | 44 (29.9) | 1 (ref) |
| **Q2** | 33 (22.4) | 0.7 (0.5, 1.1) |
| **Q3** | 35 (23.8) | 0.8 (0.5, 1.2) |
| **Q4** | 35 (23.8) | 0.7 (0.5, 1.1) |
|  |  |  |
| **Iron (µg/dL)^a,g^** | 75 (56, 91) | 0.9 (0.9, 1.0) |
| **Male** |  |  |
| **No** | 110 (74.8) | 1 (ref) |
| **Yes** | 37 (25.2) | 0.7 (0.5, 1.0) |
| **Age (years)** |  |  |
| **< 55** | 17 (11.6) | 1 (ref) |
| **55 - < 65** | 47 (32.0) | 4.4 (2.5, 7.6) |
| **≥ 65** | 83 (56.5) | 8.9 (5.3, 14.8) |
| **BMI Health** |  |  |
| **Poor** | 85 (57.8) | 1 (ref) |
| **Intermediate** | 45 (30.6) | 0.8 (0.6, 1.1) |
| **Ideal** | 17 (11.6) | 0.7 (0.4, 1.1) |
| **Pulse Pressure (mm Hg)^a,h^** | 55 (44, 73) | 1.4 (1.3, 1.5) |
| **Hypertension** |  |  |
| **No** | 26 (17.7) | 1 (ref) |
| **Yes** | 121 (82.3) | 3.3 (2.2, 5.1) |
| **Diabetes** |  |  |
| **No** | 95 (64.6) | 1 (ref) |
| **Yes** | 52 (35.4) | 2.4 (1.7, 3.3) |
| **Coronary Heart Disease** |  |  |
| **No** | 114 (77.6) | 1 (ref) |
| **Yes** | 33 (22.4) | 2.5 (1.8, 3.7) |
| **History of chronic lung disease** |  |  |
| **No** | 134 (91.2) | 1 (ref) |
| **Yes** | 13 (8.8) | 1.3 (0.8, 2.3) |
| **Spirometry Profile** |  |  |
| **Normal** | 67 (45.6) | 1 (ref) |
| **Obstructive** | 29 (19.7) | 3.6 (2.3, 5.4) |
| **Restrictive** | 51 (34.7) | 2.8 (1.9, 3.9) |
| **Left ventricle ejection Fraction (< 50%)** |  |  |
| **No** | 133 (90.5) | 1 (ref) |
| **Yes** | 14 (9.5) | 3.9 (2.4, 6.5) |
|  |  |  |
| **Hemoglobin (g/dL)^a^** | 13 (12, 13) | 0.9 (0.8, 0.9) |
| **Highly Sensitive C-Reactive Protein (mg/dL)^a^** | 0.3 (0.1, 0.7) | 1.2 (1.0, 1.4) |

^a^ Median (Quartile 1, Quartile 3)

^b^ Ferritin Deficiency: Females < 15ng/mL; Males < 30ng/mL

^c^ Females: Quartile 1 ≤ 47ng/mL; Quartile 2 > 47ng/mL – 95ng/mL; Quartile 3 > 95ng/mL – 171ng/mL; Quartile 4 > 171ng/mL

Males: Quartile 1 ≤ 110ng/mL; Quartile 2 > 110ng/mL – 182ng/mL; Quartile 3 > 182ng/mL – 294ng/mL; Quartile 4 > 294ng/mL

^d^ Prevalence Ratio expressed per 10% increase in ferritin

^e^ Iron Deficiency: Females < 30µg/dL; Males < 45µg/dL

^f^ Females: Quartile 1 ≤ 57µg/dL; Quartile 2 > 57µg/dL – 73µg/dL; Quartile 3 > 73µg/dL – 90µg/dL; Quartile 4 > 90µg/dL

Males: Quartile 1 ≤ 68µg/dL; Quartile 2 > 68µg/dL – 842µg/dL; Quartile 3 > 842µg/dL – 103µg/dL; Quartile 4 > 103µg/dL

^g^ Prevalence Ratio expressed per 10µg/dL increase in iron

^h^ Prevalence Ratio expressed per 10mmHg increase in pulse pressure
